# Supplementary material for: Profiling MHC II immunopeptidome of blood‐stage malaria reveals that cDC1 control the functionality of parasite‐specific CD4 T cells
Source: EMBO Mol Med. 2017 Sep 21;9(11):1605–21. doi: 10.15252/emmm.201708123 (PMC5666312; doi:10.15252/emmm.201708123)
Supplement: Supplementary file 1 — Expanded View Figures PDF [file EMMM-9-1605-s001.pdf]

Expanded View Figures

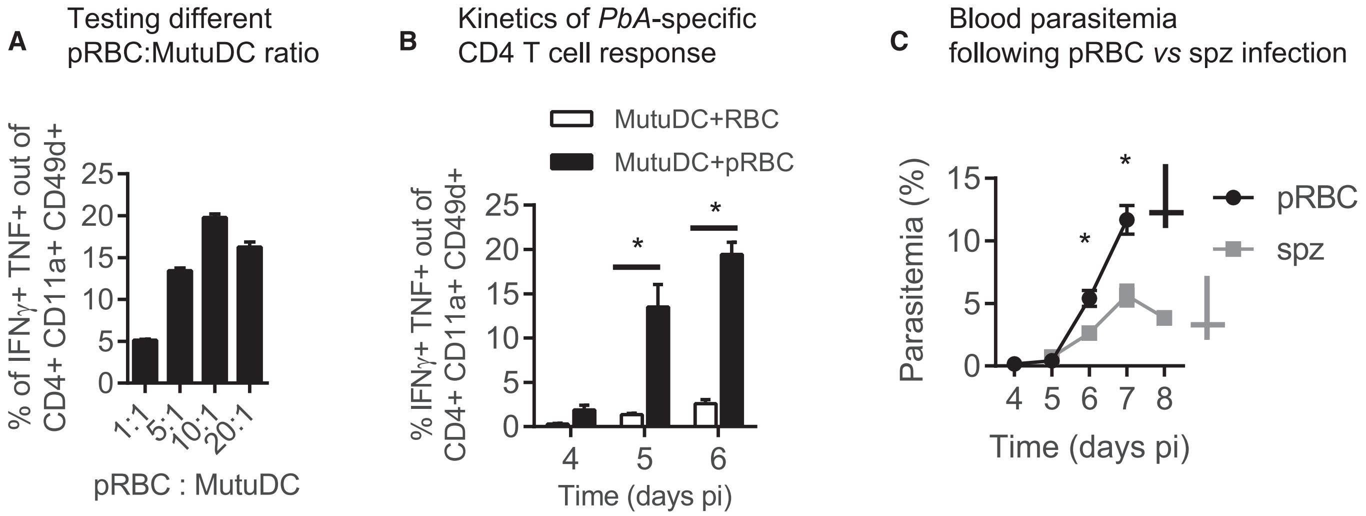

Figure EV1. *Pb*A-specific CD4 T-cell responses are maximal at day 6 post-pRBC infection.

A Proportion of CD11a$^+$ CD49d$^+$ CD4 T cells (mean ± SEM) that produce IFNγ and TNF in response to MutuDC loaded with increasing ratio of *Pb*A pRBC, analyzed at day 6 post-infection with *Pb*A pRBC. $N = 3$ mice.

B Proportion of CD11a$^+$ CD49d$^+$ CD4 T cells (mean ± SEM) that produce IFNγ and TNF in response to MutuDC loaded with *Pb*A pRBC (ratio 10:1, black bars) or uninfected RBC (white bars), after infection with *Pb*A pRBC. $N = 4$ mice per group. Asterisks show significant differences. Day 4, $P = 0.024$; Day 5, *$P = 0.003$; Day 6, *$P = 2.5 \times 10^{-5}$ by multiple unpaired *t*-tests without assuming consistent SD.

C Blood parasitemia (mean ± SEM) during infection with *Pb*A pRBC (black circles, $N = 5$ mice) or *Pb*A.GFP spz (gray squares, $N = 15$ mice). Asterisks show significant differences between the two groups. Day 4, $P = 0.14$; Day 6, *$P = 0.011$; Day 7, *$P = 0.0007$ by multiple unpaired *t*-tests without assuming consistent SD.

 © 2017 The Authors

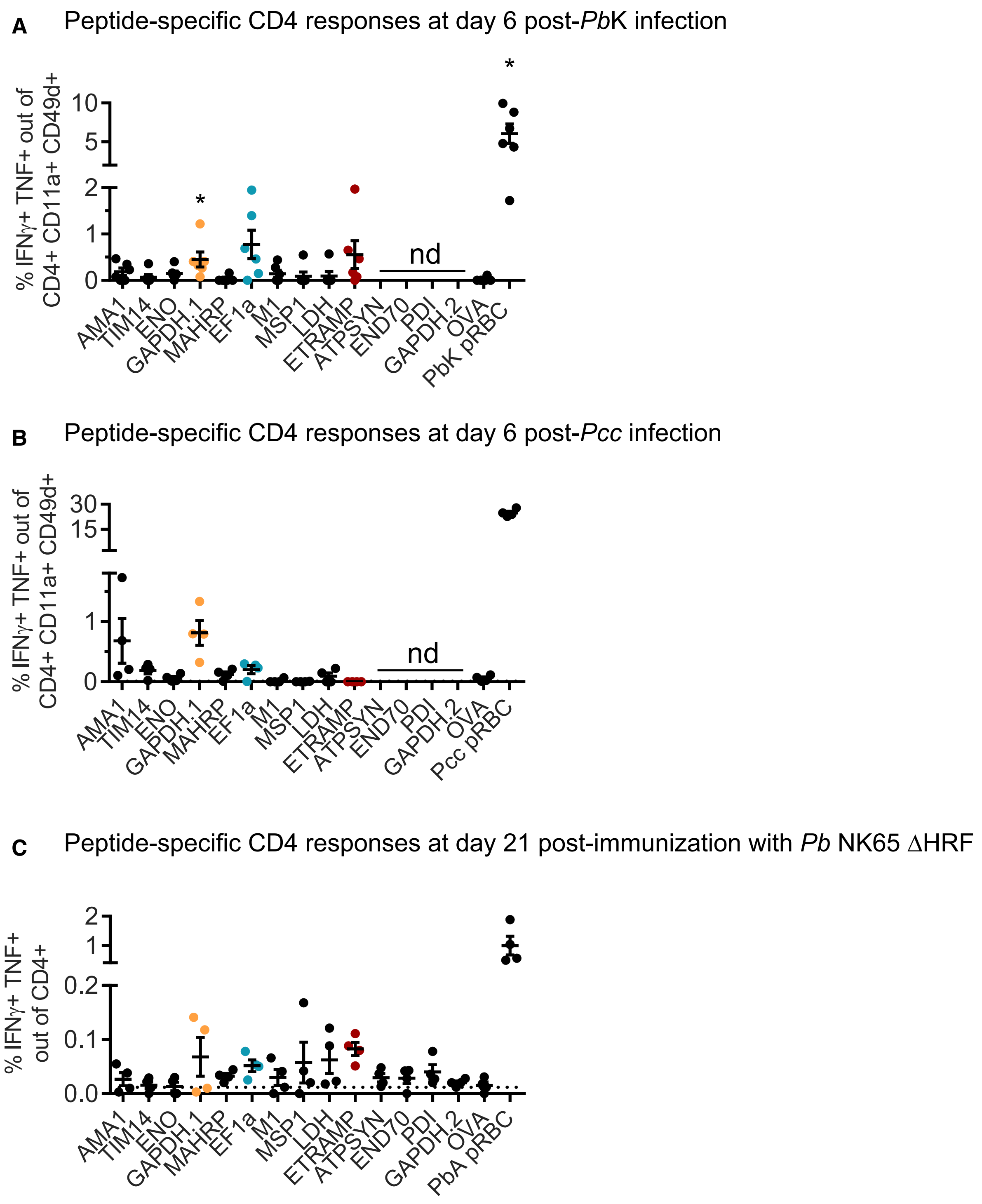

Figure EV2. Peptide-specific CD4 responses after challenge with *Pb*K, *Pcc*, or *Pb*NK65 ΔHRF.

A IFNγ/TNF-double-producing cells among activated CD4 T cells at day 6 post-infection with *Pb*K (mean ± SEM). N = 6 mice. Basal level with MutuDC alone was subtracted. Asterisks show statistical significance assessed by paired nonparametric Wilcoxon tests in comparison with OVA peptide. GAPDH.1, *P = 0.031; *Pb*K pRBC, *P = 0.031. Responses to ATPSYN, END70, PDI, and GAPDH.2 were not determined (nd). Experiments performed once.

B IFNγ/TNF-double-producing cells among activated CD4 T cells at day 6 post-infection with *Pcc* (mean ± SEM). N = 4 mice. Basal level with MutuDC alone was subtracted. Responses to ATPSYN, END70, PDI, and GAPDH.2 were not determined (nd). Experiments performed once.

C IFNγ/TNF-double-producing cells among CD4 T cells at day 21 post-challenge with genetically attenuated *Pb* NK65 ΔHRF (mean ± SEM). N = 4 mice. Basal level with MutuDC alone was subtracted. Dotted line indicates the response to OVA peptide. Experiments performed once.

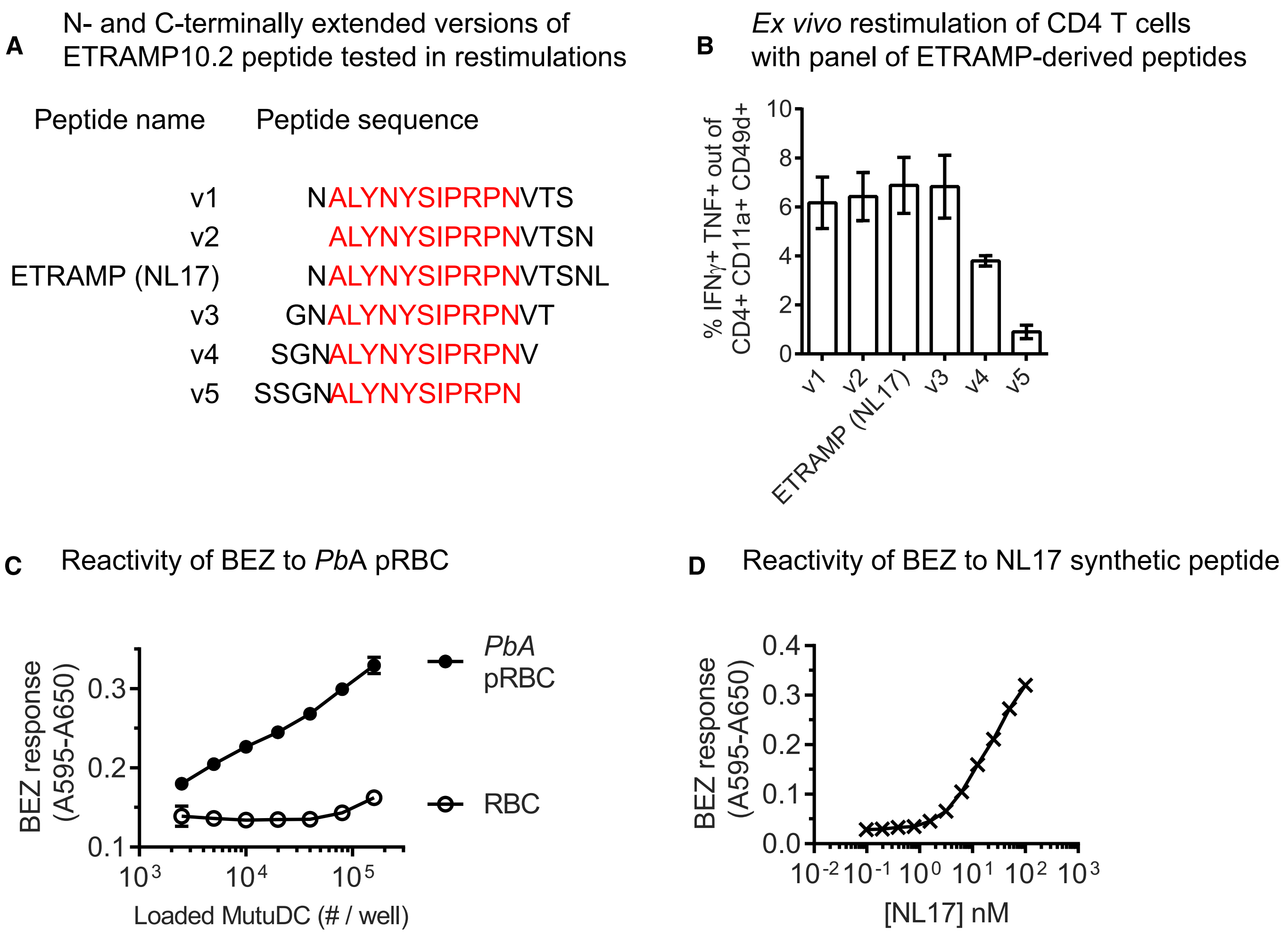

Figure EV3. ETRAMP10.2 NL17 peptide is optimal for restimulating CD4 T cells from *Pb*A-infected mice and is stimulatory to BEZ hybridomas.

A N- and C-terminally extended versions of the ETRAMP10.2 peptide used to restimulate CD4 T cells from infected mice *ex vivo*.

B IFNγ/TNF-double-producing cells among activated CD4 T cells (mean ± SEM) responding to MutuDC pulsed with the indicated peptide variants, evaluated at day 6 post-infection with *Pb*A pRBC. $N = 3$ mice.

C,D Responsiveness of BEZ hybridomas: B6-derived hybrids reactive to ETRAMP10.2 peptide and producing LacZ. β-galactosidase production by BEZ after stimulation with MutuDC loaded with RBC or *Pb*A pRBC (C) or with MutuDC incubated with serially diluted ETRAMP10.2 NL17 peptide (D). Representative of two replicates.

 © 2017 The Authors

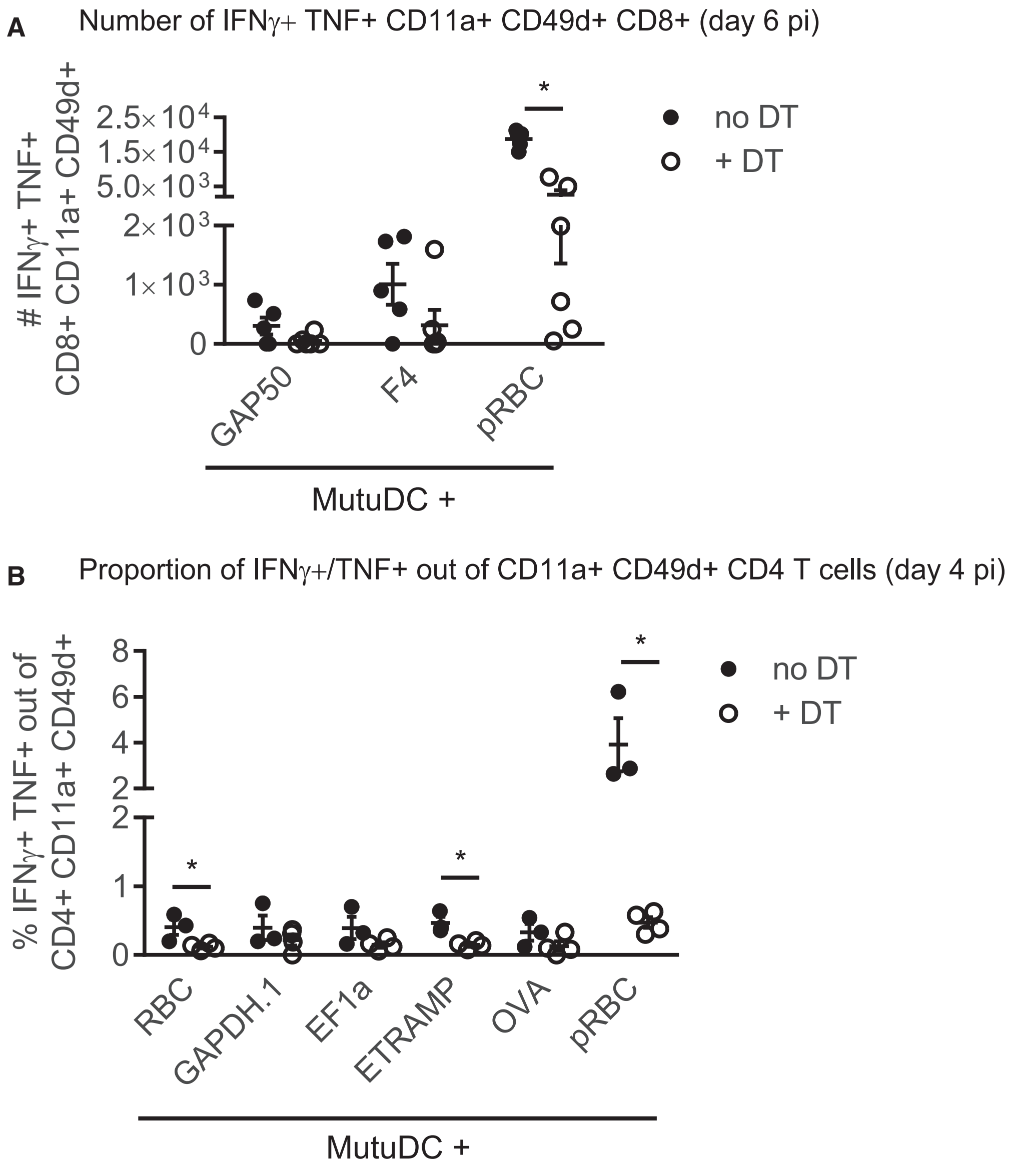

Figure EV4. cDC1 are required for the generation of parasite-specific CD8 T cells and the priming of CD4 Th1 cells during *Pb*A blood-stage malaria.

A Absolute numbers of IFNγ$^+$ TNF$^+$ CD11a$^+$ CD49d$^+$ CD8$^+$ responding to MutuDC loaded with the indicated antigens, from *Karma* mice treated (open circles) or not (black circles) with DT, analyzed at day 6 post-infection (mean ± SEM). "No DT" group: N = 5 mice, "+ DT" group: N = 6 mice. GAP50, P = 0.10; F4, P = 0.14; pRBC, *P = 6.45 × 10^{-6} by multiple unpaired t-tests without assuming consistent SD.

B Proportion of CD11a$^+$ CD49d$^+$ CD4 T cells that co-produce IFNγ and TNF in response to MutuDC loaded with the indicated antigens, from *Karma* mice treated (open circles) or not (black circles) with DT, analyzed at day 4 post-infection (mean ± SEM). "No DT" group: N = 3 mice, "+ DT" group: N = 4 mice. Asterisks show significant differences by multiple unpaired t-tests without assuming consistent SD. RBC, *P = 0.033; GAPDH.1, P = 0.36; EF1a, P = 0.14; ETRAMP, *P = 0.01; OVA, P = 0.18; pRBC, *P = 0.017.
